# Bone Geometry Is Altered by Follistatin‐Induced Muscle Growth in Young Adult Male Mice

**DOI:** 10.1002/jbm4.10477

**Published:** 2021-03-03

**Authors:** Audrey S M Chan, Narelle E McGregor, Ingrid J Poulton, Justin P Hardee, Ellie H‐J Cho, T John Martin, Paul Gregorevic, Natalie A Sims, Gordon S Lynch

**Affiliations:** ^1^ Centre for Muscle Research, Department of Anatomy and Physiology, School of Biomedical Sciences University of Melbourne Melbourne 3010 Australia; ^2^ St. Vincent's Institute of Medical Research Fitzroy 3065 Australia; ^3^ Biological Optical Microscopy Platform University of Melbourne Melbourne Australia; ^4^ Department of Medicine, St. Vincent's Hospital University of Melbourne Fitzroy 3065 Australia

**Keywords:** ANIMAL MODELS, BONE MODELING AND REMODELIN, BONE MORPHOGENETIC PROTEIN, BONE–MUSCLE INTERACTION, CELL/TISSUE SIGNALING—PARACRINE PATHWAYS, MOLECULAR PATHWAYS—REMODELING, PRECLINICAL STUDIES, SYSTEMS BIOLOGY—BONE INTERACTORS, TRANSFORMING GROWTH FACTOR β

## Abstract

The development of the musculoskeletal system and its maintenance depends on the reciprocal relationship between muscle and bone. The size of skeletal muscles and the forces generated during muscle contraction are potent sources of mechanical stress on the developing skeleton, and they shape bone structure during growth. This is particularly evident in hypermuscular global myostatin (Mstn)‐*null* mice, where larger muscles during development increase bone mass and alter bone shape. However, whether muscle hypertrophy can similarly influence the shape of bones after the embryonic and prepubertal period is unknown. To address this issue, bone structure was assessed after inducing muscle hypertrophy in the lower hindlimbs of young‐adult C57BL/6J male mice by administering intramuscular injections of recombinant adeno‐associated viral vectors expressing follistatin (FST), a potent antagonist of Mstn. Two FST isoforms were used: the full‐length 315 amino acid isoform (FST‐315) and a truncated 288 amino acid isoform (FST‐288). In both FST‐treated cohorts, muscle hypertrophy was observed, and the anterior crest of the tibia, adjacent to the tibialis anterior muscle, was lengthened. Hypertrophy of the muscles surrounding the tibia caused the adjacent cortical shell to recede inward toward the central axis: an event driven by bone resorption adjacent to the hypertrophic muscle. The findings reveal that inducing muscle hypertrophy in mice can confer changes in bone shape in early adulthood. © 2021 The Authors. *JBMR Plus* published by Wiley Periodicals LLC on behalf of American Society for Bone and Mineral Research.

## Introduction

Changes in muscle size and function can drive subsequent changes in bone shape, composition, and ultimately, bone strength. Flattened surfaces of long bones frequently correspond to areas against which the muscle belly rests, although bone ridges and crests are shaped by forces generated by muscle contraction.^(^ ^1^, ^2^ ^)^ A positive relationship between muscle growth and bone strength is particularly apparent during growth and exercise, when skeletal muscles provide mechanical stimuli to increase bone mass.^(^ ^3^, ^4^, ^5^, ^6^ ^)^ Conversely, both muscle and bone mass decline during immobilization,^(^ ^7^, ^8^ ^)^ hindlimb unloading,^(^ ^9^, ^10^ ^)^ muscular dystrophy,^(^ ^11^ ^)^ and aging.^(^ ^3^ ^)^ Hence, targeted approaches to mitigate the loss of muscle mass (sarcopenia) and bone strength concomitant with disuse, aging, and disease are needed. ^(^ ^12^, ^13^ ^)^

The TGF‐β/activin signaling pathway is crucial in the development and maintenance of both muscle and bone.^(^ ^14^, ^15^ ^)^ Soluble activin ligands, including activins A, B, and AB, myostatin (Mstn)/growth differentiation factor‐8 (GDF8) and GDF11 act through an ACVR2:ACVR1 complex and downstream Smad2/3 signaling.^(^ ^16^, ^17^ ^)^ A deficiency of the locally acting myokine Mstn leads to muscle hypertrophy in multiple species,^(^ ^17^, ^18^, ^19^, ^20^, ^21^ ^)^ including humans.^(^ ^22^ ^)^ In Mstn‐*null* mice the effect of hypermuscularity on the skeleton can be studied without confounding effects of altered physical activity.^(^ ^23^ ^)^ These mice exhibit high bone mass and strength,^(^ ^17^, ^24^, ^25^, ^26^, ^27^ ^)^ and changes in bone shape, including at sites of muscle attachment.^(^ ^24^, ^28^ ^)^

Systemic TGF‐β/activin inhibition with soluble decoy receptors (ACVR2A‐Fc^(^ ^29^, ^30^, ^31^ ^)^ or ACVR2B‐Fc^(^ ^29^, ^32^, ^33^, ^34^ ^)^) not only increased muscle mass but also increased trabecular bone volume in mice and monkeys, and further increased trabecular bone volume in Mstn‐*null* mice.^(^ ^32^ ^)^ Although the changes to bone shape, size, and mass were likely influenced by muscle hypertrophy, there is increasing evidence for direct actions of activin ligands on both osteoblasts and osteoclasts. By the inhibition of activin signaling in osteoblasts through the targeted deletion of ACVR2, the trabecular bone mass and cortical thickness in young‐adult mice were increased, partly by stimulating osteoblast differentiation, which was found in vitro.^(^ ^29^ ^)^ Furthermore, ACVR1‐*null* bone marrow macrophages (BMMs) exhibited decreased osteoclastogenesis,^(^ ^35^ ^)^ whereas BMMs, either treated with activin A^(^ ^36^, ^37^, ^38^, ^39^ ^)^ or harboring a constitutively active variant of ACVR1,^(^ ^35^ ^)^ exhibited an increase in osteoclastogenesis in vitro, indicating that activin may directly stimulate osteoclast differentiation.

Follistatin (FST) is a potent activin antagonist that sequesters soluble activin ligands^(^ ^40^, ^41^, ^42^ ^)^ and is secreted by multiple tissues, such as the liver,^(^ ^43^ ^)^ prostate,^(^ ^44^ ^)^ muscle,^(^ ^45^ ^)^ and bone.^(^ ^46^, ^47^, ^48^ ^)^ The *Fst* gene produces two protein isoforms that differ at the C‐terminus. The full‐length protein FST‐315 is the primary soluble isoform in serum.^(^ ^49^, ^50^ ^)^ In contrast, the lack of the acidic C‐terminal tail exposes the heparin‐binding sequence ^(^ ^51^ ^)^ in the truncated FST‐288 protein, allowing it to readily bind to cell surface proteoglycans and remain predominantly tissue‐bound.^(^ ^50^, ^52^ ^)^ Several studies have shown that the local or systemic overexpression of FST^(^ ^27^, ^53^, ^54^ ^)^ or FST‐derived molecules^(^ ^40^, ^55^, ^56^ ^)^ results in substantial muscle hypertrophy. FST is also produced by mesenchymal stromal cells^(^ ^47^ ^)^ and osteoblasts^(^ ^46^ ^)^; it promotes osteoblast differentiation in vitro.^(^ ^46^, ^47^ ^)^ However, in vivo studies of FST on the skeleton provide some conflicting data. Systemic FST administration in young female mice dose dependently restored bone mass after ovariectomy‐induced bone loss.^(^ ^55^ ^)^ In contrast, transgenic mice, which overexpress FST during development, exhibited smaller and weaker bones than controls.^(^ ^27^, ^57^ ^)^

The effects of rapid muscle growth on bone after the pubertal period remain unknown. To determine whether rapid muscle growth confers changes to the shape of young‐adult bone, we used recombinant adeno‐associated viral (AAV) vectors to transduce skeletal muscle with FST‐expressing constructs. The hindlimb muscles of young‐adult mice were directly injected with AAV encoding FST‐288^(^ ^53^ ^)^ or FST‐315,^(^ ^59^ ^)^ both of which have been previously validated to efficiently induce muscle hypertrophy,^(^ ^53^, ^59^ ^)^ and examined the tibia and femur after 4 weeks.

## Materials and Methods

### Generation and administration of rAAV6 vectors

cDNA constructs encoding FST‐288 and FST‐315 (Thermo Fisher Scientific) were cloned into an AAV expression plasmid consisting of a cytomegalovirus promoter/enhancer and a SV40 poly‐A region flanked by AAV2 terminal repeats as described elsewhere.^(^ ^53^, ^58^ ^)^ These plasmids were then transfected with the pDGM6 packaging plasmid into HEK293 cells, which generated type‐6 pseudotyped viral vectors that were harvested and purified as described previously.^(^ ^53^, ^58^ ^)^

### Animals

All experiments were approved by the Animal Ethics Committee of the University of Melbourne and conducted in accordance with the relevant 2004 codes of practice for the care and use of animals for scientific purposes of the National Health and Medical Council of Australia. Ten‐week‐old C57BL/6J male mice were purchased from the Animal Resource Centre and maintained at the biological research facility at the University of Melbourne. As our laboratory routinely uses male mice to study therapies for Duchenne muscular dystrophy, an X‐linked disorder affecting only males, this study was limited to male mice. Mice were housed in groups of at least three on a 12:12‐hour dark:light cycle with *ad libitum* access to standard chow diet (Specialty Feeds) and water.

The overexpression of FST in skeletal muscle was achieved by injecting nonreplicative recombinant adeno‐associated viral vector serotype 6 (AAV6)^(^ ^58^ ^)^ carrying a gene cassette of full‐length (FST‐315) or tissue‐bound (FST‐288) isoforms of FST. The injection of AAV6:FST‐288 and AAV6:FST‐315 have been previously validated to induce local muscle hypertrophy,^(^ ^53^, ^59^ ^)^ whereas AAV6:FST‐315 has also been shown to increase serum levels of FST.^(^ ^59^ ^)^ To induce muscle hypertrophy in young‐adult mice, 14‐week‐old male C57BL/6 mice (*n* = 20) were anesthetized deeply with isofluorane and were randomly selected to receive AAV6:CON (consisting of a control vector; *n* = 6), AAV6:FST‐288 (*n* = 7) or AAV6:FST‐315 (*n* = 7). AAV6 vector genomes were injected directly into the tibialis anterior (TA), extensor digitorum longus (EDL), gastrocnemius, plantaris, and soleus muscles in the left and right distal hindlimbs, where each muscle received 5 × 10^9^ vector genomes in a volume of 40 μL of Hank's balanced salt solution. After 28 days, the mice were euthanized by cervical dislocation and the hindlimb muscles and bones excised carefully and stored for later analyses. The tibias and femurs were fixed in 4% paraformaldehyde for 24 hours, rinsed in 1× PBS, and stored in 70% ethanol at 4°C. Samples were then given a nonidentifiable code before μCT scanning and subsequent analysis.

### Micro‐CT scanning and analysis

Bones were scanned in 70% ethanol using the Skyscan 1276 (Bruker microCT) with the following settings: 10‐μm voxel resolution, 0.25‐mm aluminum filter, 56‐kV voltage, 200‐μA current, 560‐ms exposure time, 0.4° rotation, and frame averaging of 2. Images were reconstructed and analyzed using NRecon (version 1.7.4.6), Dataviewer (version 1.5.6.2), CT Analyzer (CTAn; version 1.18.8.0) and CTVox (version 3.3.0 r1403). Tibial and femoral lengths were determined after scanning. Regions of interest (ROIs) were determined as described previously. ^(^ ^60^ ^)^ Trabecular bone in the tibia and femur were assessed at anatomically comparable regions. For the tibia, the ROI commenced at 3% of bone length from the growth plate and extended distally for a total of 13.5% (equivalent to 0.5–3 mm from the growth plate), and the femur ROI commenced at 7.5% from the distal growth plate and extended proximally for a total of 15%. Cortical analysis of the tibial shaft was performed between 15% and 50% of the tibial length (equivalent to 2.1‐mm and 6.2‐mm distal to the growth plate, respectively). Cortical analysis of the femur commenced at 30% from the distal growth plate and extended proximally for a total of 15%.

Multilevel Otsu thresholding was used to segregate bone into low‐, high‐, and mid‐density components as described recently. ^(^ ^61^, ^62^ ^)^ Briefly, four‐level Otsu thresholding was used across the proximal shaft of the tibia (2.1–6.2 mm, excluding trabecular bone) to categorize the gray pixels into three bone densities: low density (calibrated to 0.438–0.963 g/cm^3^ calcium hydroxylapatite [CaHA]), mid‐density (calibrated to 0.963–1.517 g/cm^3^ CaHA), and high density (calibrated to >1.517 g/cm^3^ CaHA). The lowest density quartile (0–0.438 g/cm^3^ CaHA) was excluded as background or noise.

The length of the anterior crest was measured manually, as a straight line from the tip of the crest to the endosteal surface, in each sample between 2.1–6.2 mm of the proximal tibia at every 0.2 mm using CTAn. Analyses of trabecular and cortical bone volume were completed using adaptive thresholding (mean of minimum and maximum values) in CTAn. Thresholds were as follows: 0.54 g/cm^3^ CaHA for trabecular bone in the tibia and femur, and 0.84 g/cm^3^ CaHA for cortical bone in the femur.

Radial cortical thickness was determined by initially measuring bone thickness using a custom script written in ImageJ macro using FIJI software (https://imagej.nih.gov/ij/).^(^ ^63^ ^)^ In each cross‐sectional image, a Euclidean distance map (EDM) image was generated from the inner boundary of the bone. The intensity of this EDM image is the distance from the inner boundary of the bone while uncalibrated. A straight line was then drawn from the center of the structure to the border of the image in every angle, from 0° to 359°. The thickness of the bone in each degree was calculated from the intensity of an outer boundary in EDM. For each image, the distance measurement in 360 angles was reported in a table, and the EDM image overlayed with outer boundary and centroid was saved for visual confirmation.

Anteroposterior (AP) and mediolateral (ML) diameters were analyzed in batch using a custom script written in ImageJ macro using FIJI. ^(^ ^63^ ^)^ To measure the AP and ML length of the bone, a vertical and a horizontal line was drawn from the center of mass of the structure. The center of mass was determined on the structure where the core body was retained, but the anterior crest was eliminated using an “opening (an erosion followed by a dilation)” morphological filter from the MorphoLibJ plugin.^(^ ^64^ ^)^ This preprocessing enabled the detection of the center of the mass of the structure that was more robust and less dependent on the shape variation.

Three‐dimensional reconstructions were made using CTVox that correspond to the entire data set (whole tibia) or the first 50 slices (~0.5 mm) of the ROI analyzed (cross‐sectional images). In the images where the pseudocoloring density filter was applied, the reconstructed images were not subject to postacquisition thresholding or modification; therefore, they are an unbiased representation of the relative bone density across samples. In thresholded images, a threshold of 0.54 g/cm^3^ CaHA was used for trabecular bone and 0.84 g/cm^3^ CaHA for cortical bone.

### Histology and histomorphometric analysis

Tibial samples were decalcified for 2 weeks in 15% EDTA/0.5% paraformaldehyde/PBS at 4°C, before being vertically embedded in paraffin. Transverse sections (4 μm) were cut and stained with hematoxylin (acidified) and eosin. Histomorphometry was performed using Osteomeasure (Osteometrics Inc). Endocortical perimeter (Ec.Pm) and periosteal perimeter (Ps.Pm) were defined as the linear portion of the bone at the AP or posterolateral (PL) edge of the cortex. Osteoblast perimeter (Ob.Pm/Ec.Pm, %) was determined as the percentage of the Ec.Pm that was occupied by plump, cuboidal osteoblasts. Eroded perimeter (E.Pm/Ps.Pm, %) was determined as the percentage of Ps.Pm occupied by eroded cavities.

### Statistical analysis

Data are represented as box‐and‐whisker plots with the mean and interquartile ranges, from maximum to minimum, with all the data points shown. Significant differences were identified by one‐way ANOVA followed by Tukey's multiple comparisons test in GraphPad Prism (v8.3.0.; GraphPad Inc) where *p* ≤ 0.05 was considered significant. For slice‐by‐slice analyses, each data point represents the mean of each group ± SEM, where the red bars above the graph indicate *p* ≤ 0.05 between CON and FST‐288, CON and FST‐315, and FST‐288 and FST‐315.

## Results

### Rapid muscle growth reshapes the structure of young‐adult bone

**Fig 1:**
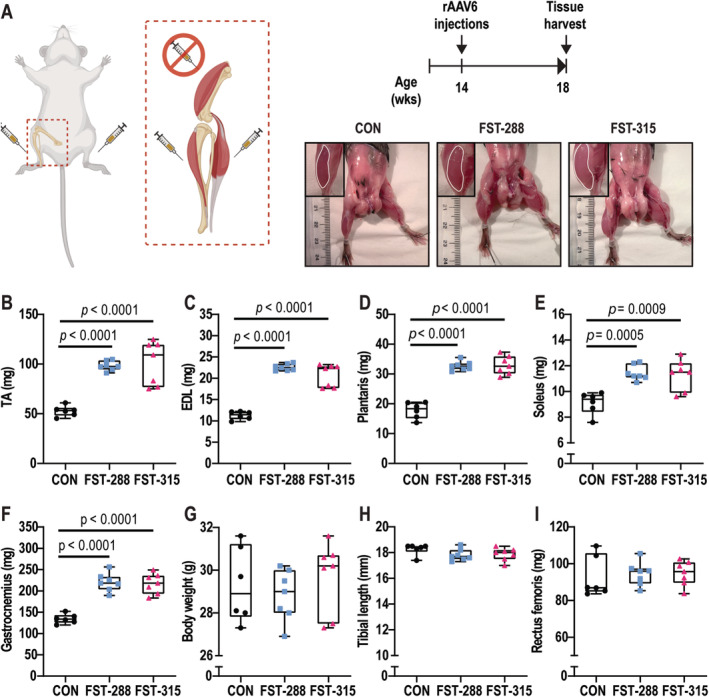
Intramuscular injections of follistatin (FST) induce muscle hypertrophy over 4 weeks. At 14 weeks of age, young male C57BL/6 mice (*n* = 20) were randomized to one of three treatment groups: AAV6:CON (adeno‐associated viral vector 6:control; *n* = 6), AAV6:FST‐288 (*n* = 7), and AAV6:FST‐315 (*n* = 7). Intramuscular injections were performed in the muscles of the distal left and right hindlimbs (tibialis anterior [TA], extensor digitorum longus [EDL], gastrocnemius, plantaris, and soleus muscles). After 4 weeks, significant muscle hypertrophy was evident (inset outlining TA muscle) (*A*). Averaged left and right muscle mass of the TA (*B*), EDL (*C*), plantaris (*D*), soleus (*E*), gastrocnemius (*F*); and final body weight (*G*), tibial length (*H*), and rectus femoris mass (*I*) of each group are shown. Data are represented as box‐and‐whisker plots with the mean and interquartile ranges from maximum to minimum, with all the data points shown. Significance was calculated using one‐way ANOVA followed by Tukey's multiple comparisons test.

Four weeks after intramuscular injections of AAV6, gross muscle hypertrophy was evident in mice overexpressing both FST‐288 and FST‐315 protein isoforms (Fig. 1*A*). Muscle mass increased by 40%–69%, which included the TA (45%), EDL (45%), and the muscles of the gastrocnemius complex (plantaris, 46%; soleus, 69%; gastrocnemius, 53%; Fig. 1*B*–*F*). There was no difference in the magnitude of muscle hypertrophy induced by FST‐288 or FST‐315 isoforms. In contrast, there were no changes in total body mass (Fig. 1*G*) or tibial length (Fig. 1*H*). Mass of the rectus femoris (part of the quadriceps muscle group) was not changed (Fig. 1*I*), confirming that the muscle hypertrophy occurred because of local injection of both FST‐288 and FST‐315.

**Fig 2:**
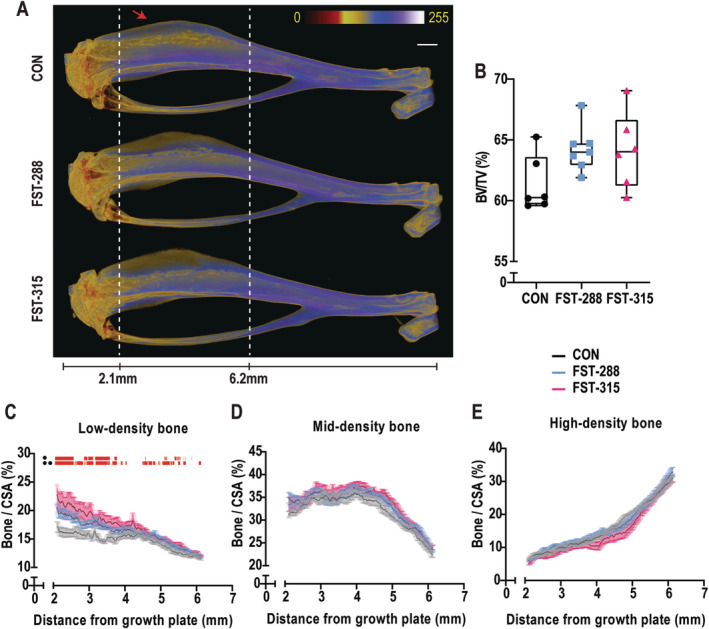
Follistatin (FST)‐induced muscle hypertrophy alters low‐density bone volume. Representative three‐dimensional reconstructions of the tibia from each cohort are shown with the application of a pseudocolor density filter to the raw acquisition files (*A*). White perforated lines mark 2.1‐mm and 6.2‐mm distal from the growth plate, with the red arrow marking the anterior crest structure. Total cortical bone volume (BV/TV) between 2.1 mm and 6.2 mm is shown as a box‐and‐whisker plot with the mean and interquartile ranges from maximum to minimum, with all the data points shown (*B*). Multilevel Otsu thresholding was used to segregate this into quantities of low (0.438–0.963 g/cm^3^ calcium hydroxylapatite [CaHA]) (*C*), mid (0.963–1.517 g/cm^3^ CaHA) (*D*), and high (>1.517 g/cm^3^ CaHA) (*E*) BMD along 2.1–6.2 mm of the tibia. Graphs are shown as the mean of each group ± SEM, where the red bars above the graph indicate *p ≤* 0.05 between control (CON) and FST‐288 or CON and FST‐315. Significance was calculated using one‐way ANOVA followed by Tukey's multiple comparisons test. Scale bar represents 1 mm.

Pseudocolored μCT images (Fig. 2*A*) showed that the bone of the tibial anterior crest adjacent to the enlarged FST‐treated TA muscle (Fig. 2*A*, red arrow) was of lower density than control bone along its full extent (from 2.1 mm to 6.2 mm from the growth plate). The total volume of cortical bone between 2.1 mm and 6.2 mm was unchanged in either of the FST cohorts when compared with the control group (Fig. 2*B*). When subcategorized into low‐, mid‐ and high‐density bone,^(^ ^61^ ^)^ the FST‐288 and FST‐315 cohorts exhibited a greater volume of low‐density bone in the crest than controls, particularly in the region closest to the growth plate, whereas there was no difference between FST‐288 and FST‐315 cohorts (Fig. 2*C*). There were no changes in the volumes of mid‐ or high‐density bone (Fig. 2*D*,*E*). This indicates that FST production by the muscles surrounding the tibia increased the amount of low‐density bone along the tibial anterior crest without changing total bone volume, suggesting a change in shape.

**Fig 3:**
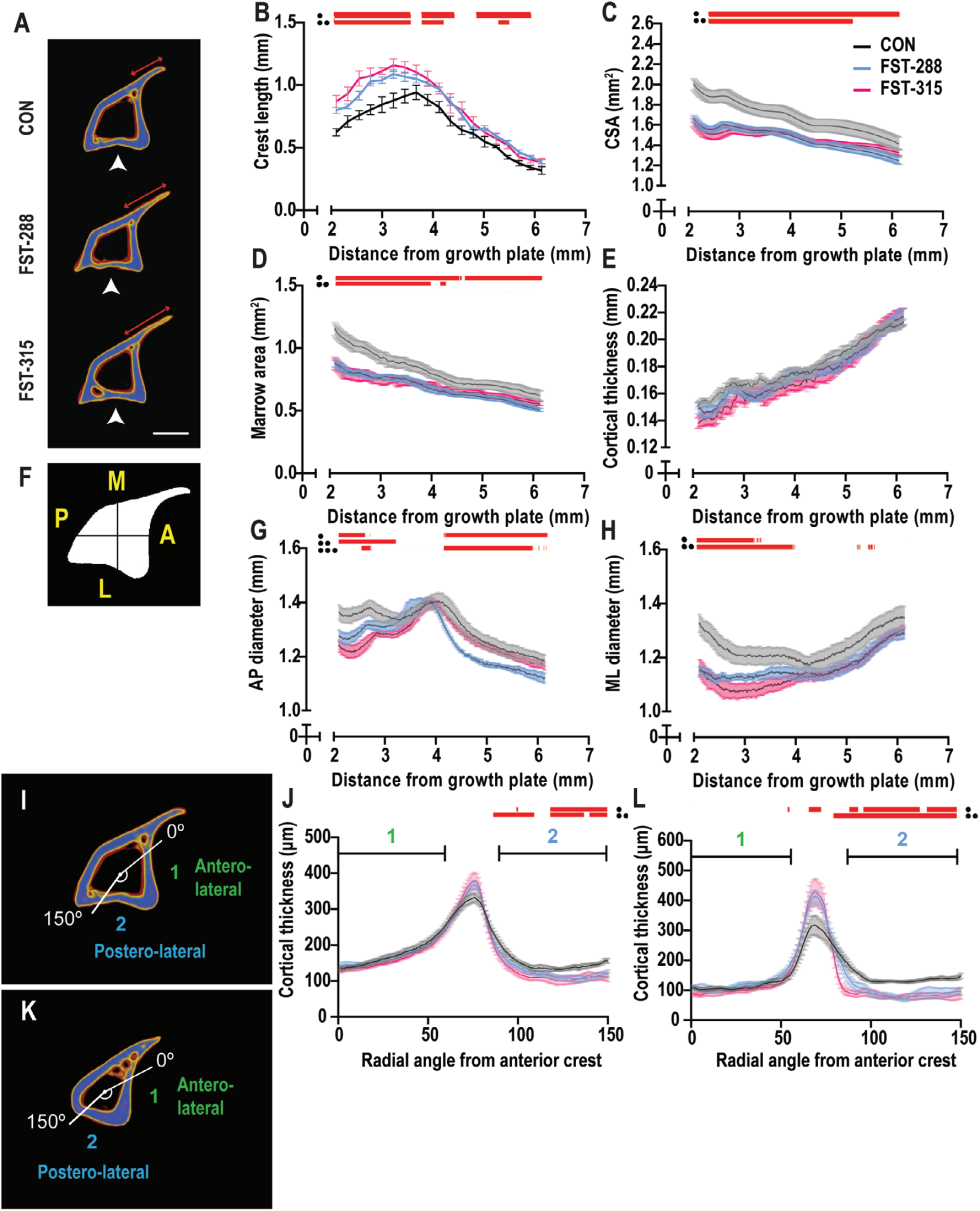
Follistatin (FST)‐induced muscle hypertrophy alters geometric dimensions of the tibial cortex. Representative three‐dimensional reconstructions of the tibia at 3.5‐mm distal from the growth plate are shown with the application of a pseudocolor density filter applied to the raw acquisition files (*A*). White arrowhead indicates region of cortical thinning, red arrow marks the length of the anterior crest. Analysis of the structural dimensions of the tibia spanning across 2.1–6.2 mm included the length of the anterior crest (*B*), cross‐sectional area (CSA; *C*), marrow area (*D*), mean cortical thickness (*E*), anteroposterior (AP; *F*,*G*), and mediolateral diameters (*H*). Cortical thickness at the anterolateral (region 1) and posterolateral (region 2) was determined at 3.5 mm (*I*,*J*) or 5.2 mm (*K*,*L*) distal to the growth plate. These measurements were graphed relative to the anterior crest (0°) and extended radially for 150°. All graphs represent the means of each group ± SEM, where the red bars above the graph indicate *p ≤* 0.05 between control (CON) and FST‐288, CON and FST‐315, and FST‐288 and FST‐315. Significance was calculated using one‐way ANOVA followed by Tukey's multiple comparisons test. Scale bar represents 1 mm.

We then analyzed the shape of the proximal half of the tibia (2.1–6.2 mm from the growth plate) because the increase in low‐density bone occurred predominantly in this region. The anterior crest was consistently longer in both FST cohorts (Fig. 3*A*,*B*). To ascertain whether this was the result of the central cortical shell receding inward toward the central axis or because of extended growth of the crest, we measured the transverse dimensions of the tibia. The total cross‐sectional area (CSA; Fig. 3*C*) and marrow area (Fig. 3*D*) were both significantly smaller in FST cohorts compared with control, along the length of the proximal tibia, with no difference in mean cortical thickness (Fig. 3*E*). Further analysis revealed that the smaller CSA observed in both of the FST cohorts was the result of smaller AP and ML diameters (Fig. 3*G*,*H*). Although this occurred in both groups in the region closest to the growth plate, only the expression of FST‐288 caused a further change in the diaphysis (between 4.5 mm and 6 mm). Altogether, this confirms that muscle hypertrophy, induced by local FST overexpression in muscle, altered the dimensions of the tibial bone and resulted in a narrower shaft.

Although mean cortical thickness was not modified by FST (Fig. 3*E*), there appeared to be a localized thinning of cortical bone in the FST‐288 and FST‐315 cohorts that was not observed in controls (Fig. 3*A*, white arrowheads). To confirm this, we measured the cross‐sectional thickness of the cortical bone radially (0–360°) at two positions: (i) at the region exhibiting prominent narrowing of the tibial shaft (a distance of 3.5‐mm distal to the growth plate; Fig. 3*I*,*J*), and (ii) at the diaphysis (5.2‐mm distal to the growth plate; Fig. 3*K*,*L*). From these measurements, we limited our analyses to include the bone adjacent to the TA and EDL muscles (region 1, the anterolateral [AL] edge) and the bone opposed by the gastrocnemius muscle complex (region 2, the posterolateral edge). The thickness of cortical bone at the AL surface was not modified at either site (Fig. 3*I*–*L*, region 1), but cortical bone at the PL edge was thinner. This was observed in both FST cohorts (Fig. 3*I*–*L*, region 2), suggesting that bone shape was modified at the surface adjacent to the gastrocnemius muscle complex.

**Fig 4:**
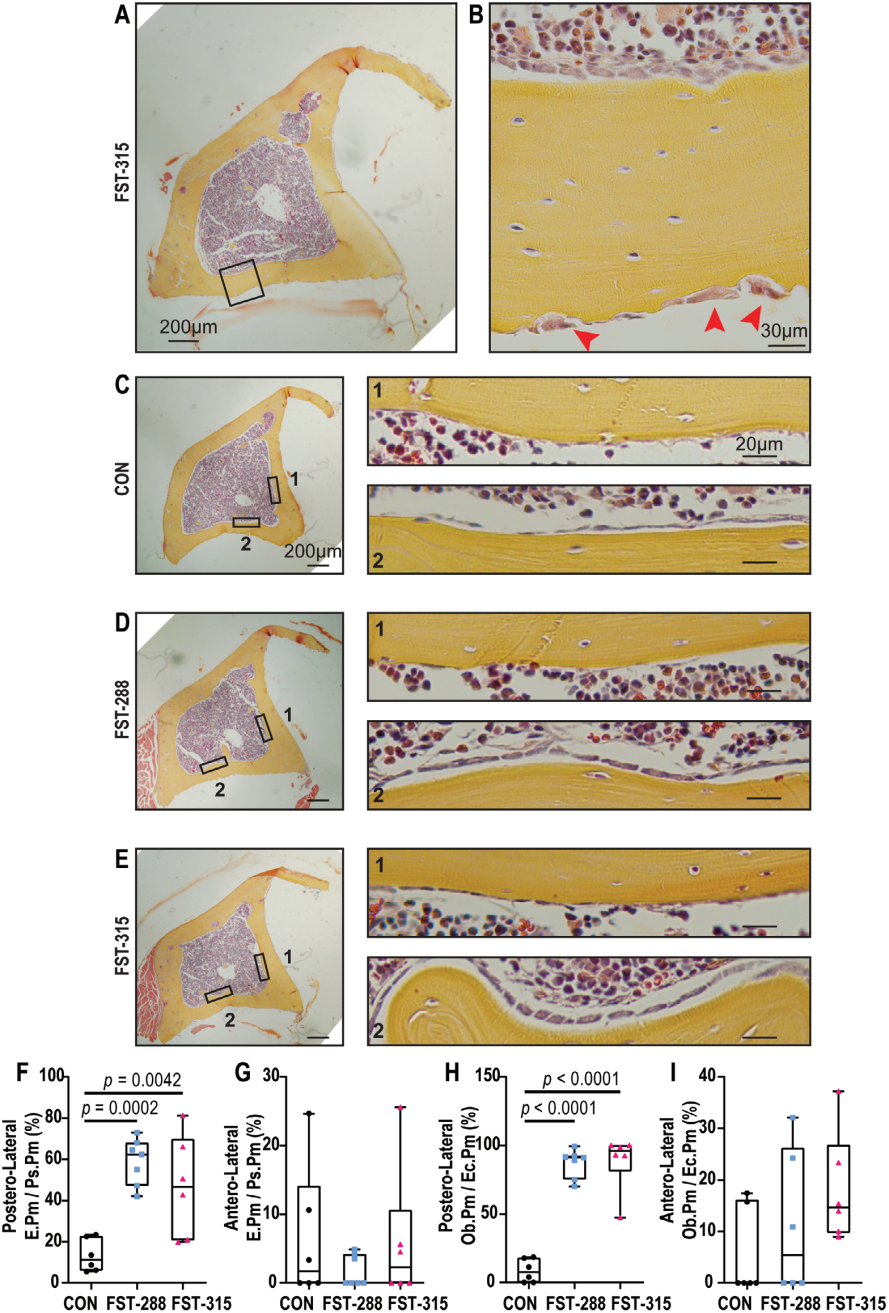
Muscle hypertrophy drives “bone drift” at the posterolateral cortex. Representative images of hematoxylin and eosin‐stained cross section of FST‐315 (follistatin 315). Scale bar represents 200 μm (*A*). Magnified inset depicting osteoblasts at the endocortical surface and osteoclasts at the periosteal surface (red arrowheads). Scale bar represents 30 μm (*B*). Representative images of control (CON) (*C*), FST‐288 (*D*), and FST‐315 (*E*). scale bar represents 200 μm. Magnified images of the endocortical surface of the anterolateral (1) and posterolateral cortex (2) shown adjacent to full‐sized images. Scale bar represents 20 μm. Histomorphometric analyses of eroded surface per periosteal perimeter (E.Pm/Ps.Pm) at the posterolateral (*F*) and anterolateral (*G*) cortex, and osteoblast perimeter per endocortical perimeter (Ob.Pm/Ec.Pm) at the posterolateral (*H*) and anterolateral (*I*) cortex. Data are represented as box‐and‐whisker plots with the mean and interquartile ranges, from maximum to minimum, with all the data points shown. Significance was calculated using one‐way ANOVA followed by Tukey's multiple comparisons test.

To determine the mechanism by which localized thinning of the cortical bone occurred, we examined the cortical bone histologically (Fig. 4). An abnormally high level of bone resorption was observed at the periosteal surface adjacent to the enlarged gastrocnemius muscle complex (shown is an example from the FST‐315 cohort). This was indicated by the presence of osteoclasts and Howship's lacunae, the latter being rarely seen in murine bone under physiological conditions (Fig. 4*A*,*B*, red arrowheads). Furthermore, on the opposite side of the cortex undergoing resorption, we observed an uninterrupted monolayer of plump osteoblasts on the endocortical side (Fig. 4*B*). This was observed in both FST cohorts (Fig. 4*C*–*E*, inset 2), and was limited to the PL cortex (inset 2), with few plump osteoblasts being observed at the AL cortex in all three groups (inset 1). Quantitation confirmed significantly greater eroded bone surface at the PL periosteal surface, adjacent to the gastrocnemius complex, but not the AL surface adjacent to the TA and EDL (Fig. 4*F*,*G*). This was matched with a significantly greater osteoblast surface, again on the PL endocortical surface, but not the AL endocortical surface (Fig. 4*H*,*I*). This suggests that overexpression of FST in the gastrocnemius muscle complex induced cortical drift in the adjacent cortical bone by stimulating localized bone resorption on the adjacent periosteum and bone formation on the endocortical surface.

### Greater trabecular bone mass observed in the tibia and femur in mice injected with AAV:FST‐315

**Fig 5:**
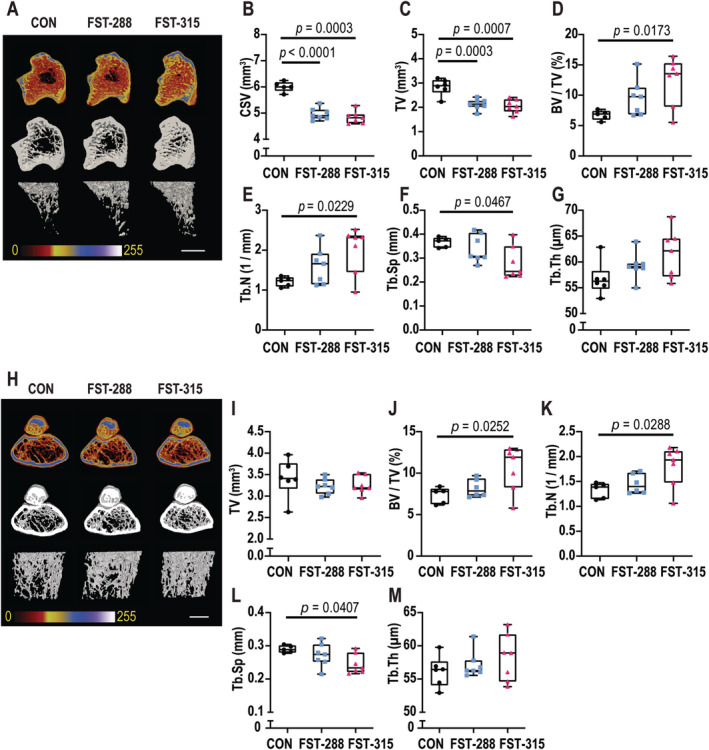
Greater trabecular bone mass observed in tibia and femur following expression of follistatin (FST). Representative three‐dimensional reconstructions of the proximal tibia are shown with a pseudocoloring density filter applied to the raw acquisition files (*A*, top row) or after binary thresholding (*A*, middle and bottom rows). Trabecular bone parameters include cross‐sectional volume (CSV; *B*), tissue volume (TV; *C*), bone volume ratio (BV/TV; *D*), trabecular number (Tb.N; *E*), trabecular separation (Tb.Sp; *F*), and trabecular thickness (Tb.Th; *G*). Representative three‐dimensional reconstructions of the distal femur are shown with a pseudocoloring density filter applied to the raw acquisition files (*H*, top row) or after binary thresholding (*H*, middle and bottom rows). Trabecular bone parameters include TV (*I*), BV/TV (*J*), Tb.N (*K*), Tb.Sp (*L*), and Tb.Th (*M*). Data are represented as box‐and‐whisker plots with the mean and interquartile ranges, from maximum to minimum, with all the data points shown. Significance was calculated using one‐way ANOVA followed by Tukey's multiple comparisons test. Scale bar represents 1 mm. CON = control.

We next determined the effects of FST overexpression in the lower limb musculature on trabecular bone mass. Consistent with the observed narrowing of the tibial shaft associated with muscle hypertrophy, both FST‐288 and FST‐315 tibias exhibited a smaller cross‐sectional volume (Fig. 5*B*) and lower total tissue volume (Fig. 5*C*) in the tibial metaphysis. Trabecular bone volume (Fig. 5*D*) and trabecular number (Fig. 5*E*) were significantly greater in the presence of muscles expressing full‐length FST when compared with control. The FST‐288 cohort showed no significant difference compared with either control or FST‐315.

To further investigate whether these observations were not because of muscle hypertrophy directly and change in bone shape, we also analyzed trabecular bone mass in the distal femur, a bone not adjacent to the hypertrophied muscle. There was no detectable change in the morphology of the femur (Supplementary Information Fig. S1; Fig. 5*H*). This supports a conclusion that geometric alterations to the tibia were secondary to hypertrophy of adjacent muscle. Similar to the observations in the proximal tibia, the expression of full‐length FST resulted in significantly greater trabecular bone volume (Fig. 5*J*) and trabecular number (Fig. 5*K*) in the distal femur when compared with control. The FST‐288 cohort was not significantly different from either the control or the FST‐315 cohort.

## Discussion

Here we have observed that muscle hypertrophy, induced by expression of either full‐length or truncated FST, induced a change in bone shape in young‐adult bone, specifically an extension of the anterior crest and narrowing of the cortex. We propose that the localized change in bone shape at the tibial cortex occurred in response to muscle hypertrophy and was driven by bone modeling, specifically “bone drift.”

Bone modeling and remodeling serve distinct purposes to maintain bone strength. Bone remodeling maintains normal shape and is coordinated by a series of events to facilitate the localized removal of damaged bone with the replacement of newly synthesized matrix on the same bone surface.^(^ ^65^, ^66^ ^)^ In contrast, bone modeling facilitates a change in shape by stimulating osteoblast and osteoclast activities on different bone surfaces. Coordination of these modeling processes across the cortical bone enables shape change^(^ ^67^, ^68^ ^)^ or bone drift^(^ ^69^ ^)^; this facilitates transverse growth of bone and adjustments to curvature in response to growth and mechanical cues.^(^ ^70^ ^)^

**Fig 6:**
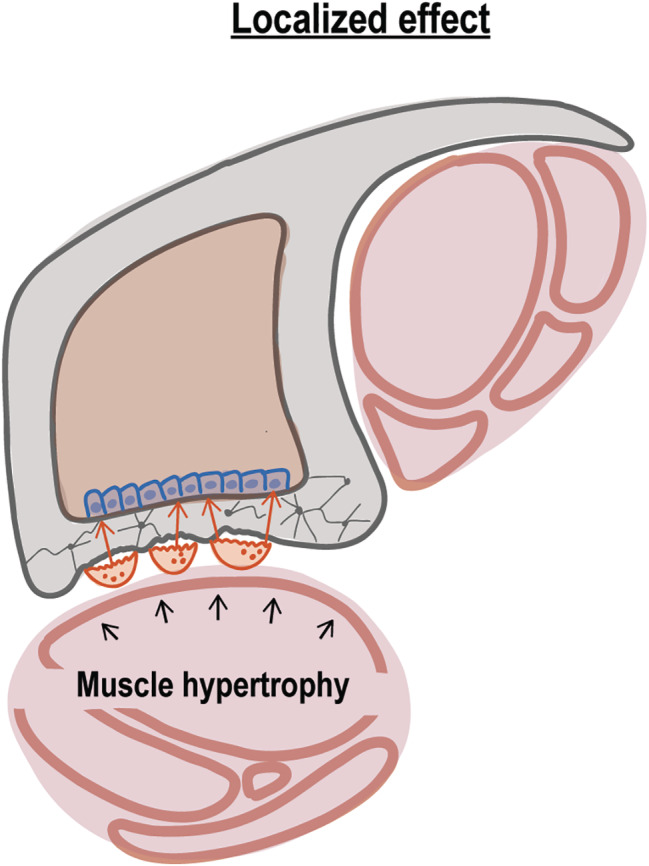
Proposed localized effect of follistatin‐induced muscle hypertrophy on bone modeling. Follistatin‐induced muscle hypertrophy exerts large pressures at the surface of the cortical bone and facilitates bone drift by inducing osteoclast resorption at the periosteum and activation of osteoblasts on the opposing side.

In the present study, thinning of the PL cortex was characterized by bone resorption on the periosteal surface adjacent to the hypertrophic muscle. Because this was only observed adjacent to the hypertrophied muscle, we conclude that this was a direct effect of muscle growth. We suggest that the localized bone resorption, observed adjacent to the large gastrocnemius muscle complex, occurred in response to compressional forces exerted by these muscles on the bone surface (Fig. 6). Such an effect of muscle force on bone resorption is consistent with early studies confirming that compressive forces generated externally^(^ ^71^ ^)^ or in a tail‐bending model^(^ ^72^ ^)^ induced resorption at the periosteal surface. This is also similar to the underlying principle of orthodontic tooth movement, where a zone of “compression” generated on the alveolar bone in the direction of movement leads to focal necrosis and subsequent release of chemoattractants and cytokines by periodontal ligament and inflammatory cells that lead to osteoclast formation, such as IL‐6 and TNF‐α.^(^ ^73^, ^74^ ^)^ Similarly, in our model, compressive forces exerted by the gastrocnemius muscle complex were sufficient to induce bone resorption at the periosteal bone surface; however, the cytokines responsible for inducing bone resorption and the cells that release them in response to compressive force remain to be identified. Furthermore, although the TA and EDL muscles were also hypertrophic and a shape change was observed, resorption was not detected on their adjacent periosteal surface. We attribute this to the region where the transverse sections were cut, which coincided with the site 3–3.5‐mm distal from the growth plate. At this location, FST‐mediated muscle hypertrophy resulted in a smaller ML diameter (Fig. 3*H*) but not AP diameter (Fig. 3*G*). Therefore, this most likely explains the lack of bone modeling observed at the AL cortex. It should be noted that we cannot, at this stage, experimentally test any separate action of FST independent of muscle hypertrophy on bone shape at this site because Mstn‐KO mice also respond to FST with a further increase in hypertrophy.^(^ ^53^ ^)^

The increased resorption induced by muscle hypertrophy was paired with increased formation of osteoblasts on the opposite endocortical surface. The communication pathway between these two processes across the cortical bone is not known, although two similar processes have been proposed. In a recent study,^(^ ^75^ ^)^ compressive forces exerted by the enlarging brain corresponded to regions of resorption at the endocranial surface, similar to our observations on the periosteum adjacent to hypertrophic muscle. On the opposite side of the cranial bone, regions of tensile stress predicted by finite element analysis modeling correlated with areas of bone formation on the ectocranial surface. This “*trans*‐pairing” of resorption and formation across the calvarial bone^(^ ^75^ ^)^ requires coordinated communication between osteoclasts and osteoblasts through the bone marrow space that separates the two cranial bone layers. In our model, the coordination of resorption with formation occurs directly through cortical bone. Such osteoclast–osteoblast communication across cortical bone during bone modeling has been attributed, in part, to osteoclast‐derived “osteotransmitters.”^(^ ^66^, ^76^ ^)^ These were first described in a model with deletion of gp130 in mature osteoclasts and impaired periosteal bone formation, where osteoclast resorptive capacity was normal, but their ability to communicate with bone‐forming osteoblasts on the opposing cortical bone surface was compromised.^(^ ^76^ ^)^ The identity of these signaling factors remains to be determined, and it is not known to what extent they may overlap with established osteoclast‐derived “coupling factors” that facilitate the coupling of bone resorption with bone formation during bone remodeling,^(^ ^66^ ^)^ such as sphingosine‐1‐phosphate or cardiotrophin‐1.^(^ ^77^ ^)^ Although earlier work has suggested osteotransmitters can be released independently of osteoclast's resorptive activity, they may also, just like coupling factors, be released from the bone matrix and could include TGF‐β and BMP2,^(^ ^78^ ^)^ which would require their transport through the lacunocanalicular network of cortical bone. This may also involve mechanically sensitive proteins like periostin^(^ ^79^ ^)^ and sclerostin,^(^ ^80^ ^)^ as well as mechanical cues generated by changes in cortical structure detected by osteocytes within the cortex.

We also observed longer tibial anterior crests concomitant with muscle hypertrophy induced by both FST isoforms. This was partly because of the cortical shell receding inward as described, but the high proportion of bone with low mineral density in this region and the eroded bone surface immediately adjacent to the TA muscle (Supplementary Information

Fig. S2), suggests the bony ridge was actively lengthened in response to muscle hypertrophy. Bony ridge expansion has been described at muscle attachment sites on the humerus^(^ ^24^ ^)^ and femur^(^ ^25^, ^28^ ^)^ in Mstn‐*null* mice in response to frequent stretching of muscle during skeletal development. In contrast, in our model of muscle hypertrophy during early adulthood, the anterior crest was lengthened at the region where the CSA of the TA muscle belly was greatest. The TA muscle and anterior crest are not connected via tendon, but through direct fusion of the connective tissues surrounding the muscle (epimysium) and bone (periosteum). The lengthening of the anterior crest may therefore have been stimulated by tensile strains on the epimysium in response to muscle hypertrophy being propagated to the periosteum at the edge of the anterior crest. This is based on previous studies documenting bone formation at regions of tensile strain in tooth movement^(^ ^81^, ^82^ ^)^ and tail‐bending models.^(71)^ Although the site at which bone‐shape change occurs differs between development and adulthood, our study clearly shows that muscle hypertrophy during early adulthood can change bone shape well after skeletal development is complete.

In addition to the effects of muscle hypertrophy on bone shape, induced FST‐315 expression was associated with greater trabecular bone volume in both tibia and femur. The increased trabecular bone mass in FST‐315 mice occurred at the distal femur, which was not adjacent to the hypertrophic muscle. This suggests FST action on trabecular bone volume may be independent of muscle hypertrophy. This is supported by work showing that overexpression of FST‐315 in muscle, using the same methods employed here, increases serum levels of FST^(^ ^59^ ^)^; we were not able to confirm or refute this in the present study. The increased trabecular bone volume is consistent with several earlier studies reporting increased trabecular bone mass with systemic inhibition of activin signaling by soluble FST^(^ ^47^, ^55^ ^)^ or activin decoy receptors (ACVR2A/2B‐Fc).^(^ ^29^, ^31^, ^32^, ^34^, ^55^, ^83^ ^)^ We cannot exclude or confirm whether FST‐288 had a similar effect because the trabecular structure of these mice was not significantly different to either control or FST‐315.

Collectively, this study reports changes to the structure of cortical bone as the result of FST‐induced muscle hypertrophy. Our findings clearly show hypertrophied muscles in the young‐adult skeleton induce bone resorption at the adjacent periosteum, an event paired with osteoblast activation at the endosteal bone surface (Fig. 6). Furthermore, overexpression of FST leads to greater trabecular bone volume in both the tibia and femur, an event that may reflect the local release of FST into nearby tissues. Together, these results confirm a substantial influence of muscle hypertrophy on bone shape in the young‐adult skeleton.

## Conflict of Interest

The authors declare no competing interests.

### Peer review

The peer review history for this article is available at https://publons.com/publon/10.1002/jbm4.10477.

## Supporting information


**Figure S1**: **FST‐induced hypertrophy of lower hindlimb muscles does not affect femur geometry.** Representative three‐dimensional reconstructions of the femoral diaphysis are shown with the application of a pseudocolor density filter applied to the raw acquisition files (A, top, bottom rows) or after binary thresholding (middle row). No differences were observed in cross‐sectional area (B), tissue area (C), cortical area (D), marrow area (E), cortical thickness (F) or mean moment of inertia (G) between control, FST‐288 and FST‐315 cohorts. Data are represented as box‐and‐whisker plots with the mean and interquartile ranges from maximum to minimum, with all the data points shown. Significance was calculated using one‐way ANOVA followed by Tukey's multiple comparisons test.

**Figure S2**: **FST‐induced muscle hypertrophy stimulated bone modelling at the tibial anterior crest.** Transverse sections of the tibial anterior crest at 3.5 mm distal to the growth plate from the control (CON, top) or muscle hypertrophy (FST‐288, bottom) cohort. Sections were stained with haematoxylin and eosin. An irregular bone surface was observed directly adjacent to the hypertrophied TA muscle (orange dotted outline), in the FST‐288 sample, with the expansion of the periosteum on the opposite side of the anterior crest. Scale bar represents 50 μm.
