# Supplementary figures and images for: Bone Geometry Is Altered by Follistatin‐Induced Muscle Growth in Young Adult Male Mice
Source: JBMR Plus. 2021 Mar 3;5(4):e10477. doi: 10.1002/jbm4.10477 (PMC8046154; doi:10.1002/jbm4.10477)

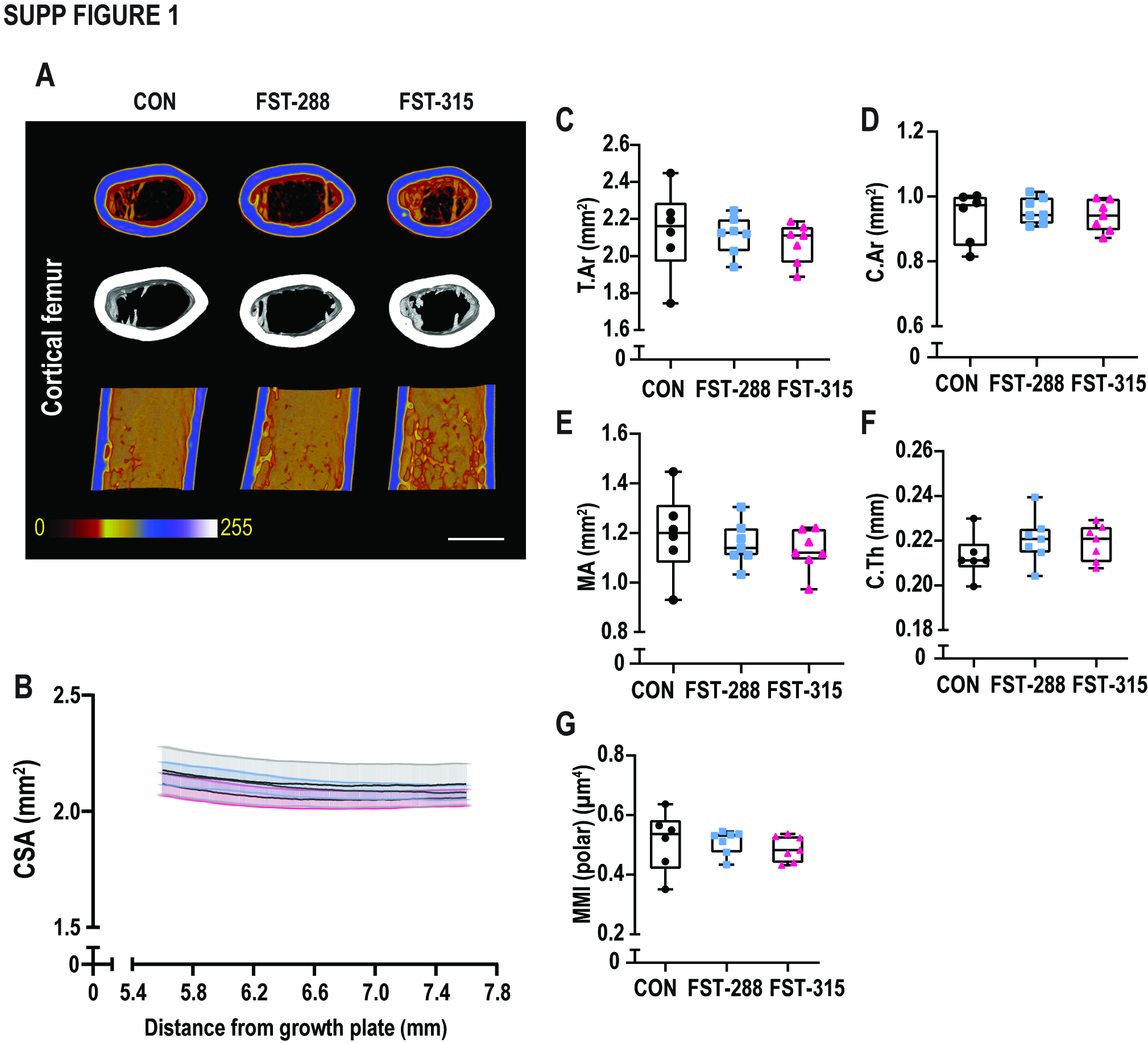

Supplement: Supplementary file 1 — Figure S1: FST‐induced hypertrophy of lower hindlimb muscles does not affect femur geometry. Representative three‐dimensional reconstructions of the femoral diaphysis are shown with the application of a pseudocolor density filter applied to the raw acquisition files (A, top, bottom rows) or after binary thresholding (middle row). No differences were observed in cross‐sectional area (B), tissue area (C), cortical area (D), marrow area (E), cortical thickness (F) or mean moment of inertia (G) between control, FST‐288 and FST‐315 cohorts. Data are represented as box‐and‐whisker plots with the mean and interquartile ranges from maximum to minimum, with all the data points shown. Significance was calculated using one‐way ANOVA followed by Tukey's multiple comparisons test. [file JBM4-5-e10477-s001.tif]

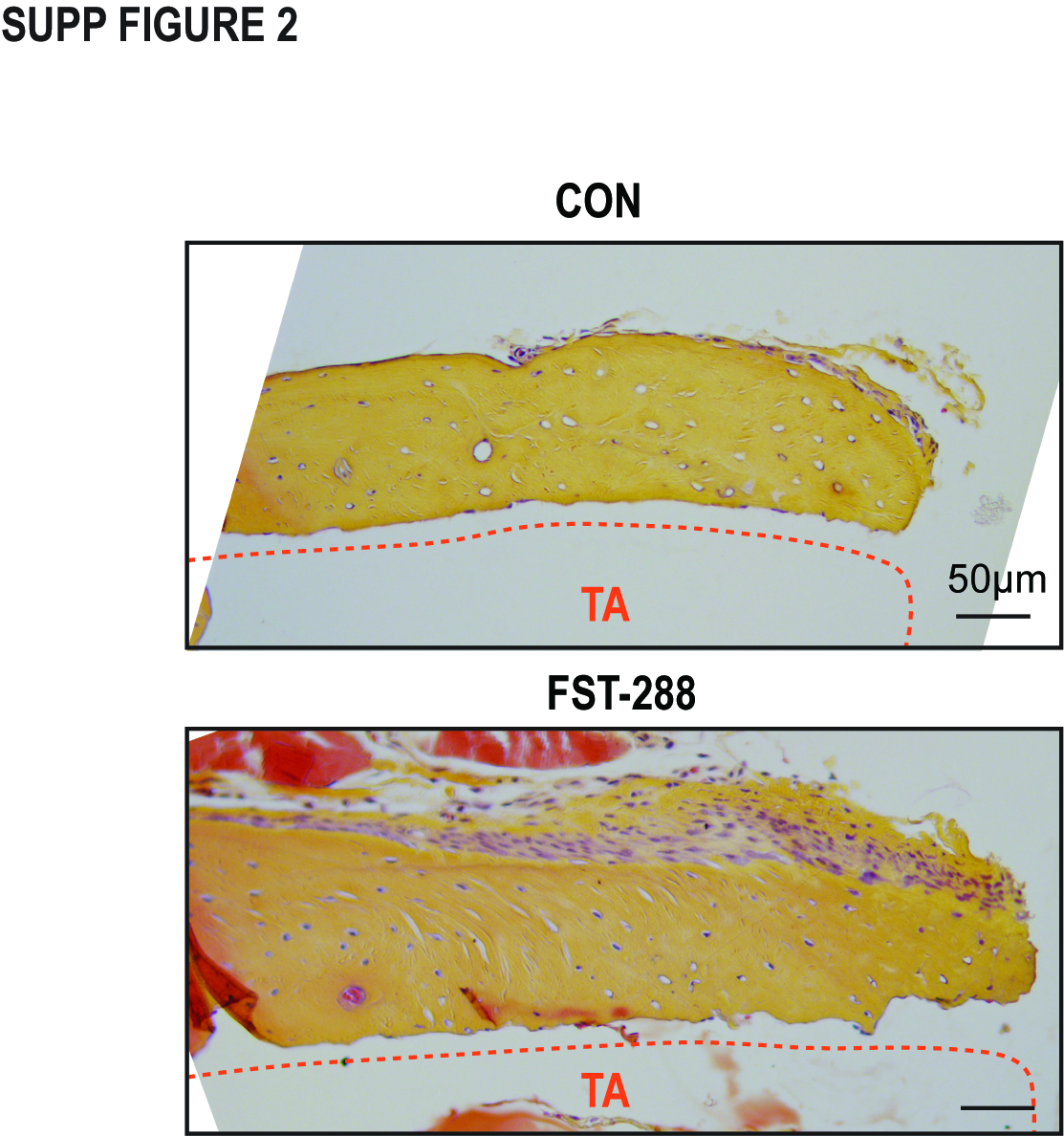

Supplement: Supplementary file 2 — Figure S2: FST‐induced muscle hypertrophy stimulated bone modelling at the tibial anterior crest. Transverse sections of the tibial anterior crest at 3.5 mm distal to the growth plate from the control (CON, top) or muscle hypertrophy (FST‐288, bottom) cohort. Sections were stained with haematoxylin and eosin. An irregular bone surface was observed directly adjacent to the hypertrophied TA muscle (orange dotted outline), in the FST‐288 sample, with the expansion of the periosteum on the opposite side of the anterior crest. Scale bar represents 50 μm. [file JBM4-5-e10477-s002.tif]
